# Examination of Beam Theories for Buckling and Free Vibration of Functionally Graded Porous Beams

**DOI:** 10.3390/ma17133080

**Published:** 2024-06-22

**Authors:** Shuaishuai Wu, Yilin Li, Yumei Bao, Jun Zhu, Helong Wu

**Affiliations:** 1 College of Mechanical Engineering, Zhejiang University of Technology, Hangzhou 310014, China; 2 College of Mechanical Engineering, Zhijiang College of Zhejiang University of Technology, Shaoxing 312030, China

**Keywords:** functionally graded porous beam, buckling, free vibration, beam theory, differential quadrature method

## Abstract

This paper examines the accuracy and effectiveness of various beam theories in predicting the critical buckling loads and fundamental frequencies of functionally graded porous (FGP) beams whose material properties change continuously across the thickness. The beam theories considered are classical beam theory (CBT), first-order shear deformation beam theory (FSDBT), third-order shear deformation beam theory (TSDBT), and the broken-line hypothesis-based shear deformation beam theory (BSDBT). Governing equations for those beam theories are formulated by using the Hamilton’s principle and are then solved by means of the generalised differential quadrature method. Finite element simulation solutions are provided as reference results to assess the predictions of those beam theories. Comprehensive numerical results are presented to evaluate the influences of the porosity distribution and coefficient, slenderness ratio, and boundary condition on the difference between theoretical predictions and simulation results. It is found that the differences significantly increase as the porosity coefficient rises, and this effect becomes more noticeable for the rigid beam with a smaller slenderness ratio. Nonetheless, the results produced by the BSDBT are always the closest to simulation ones. The findings in this paper will contribute to the establishment of more refined theories for the mechanical analysis of FGP structures.

## 1. Introduction

Porous materials are a class of substances that contain numerous interconnected or enclosed pores or voids inside, with the key features of high porosity, low density, large specific surface area, and high specific strength. These characteristics contribute to their lightweight, good sound and heat insulation, high permeability, and energy adsorption capacity, making porous materials suitable for widespread applications in aerospace, automotive, construction, and other industries [1,2,3]. The development of porous materials has therefore become an active area of research, with scientists exploring new methods to prepare and modify porous materials with desired properties for specific applications.

One of recent advances in porous materials is the functionally graded porous materials (FGPMs), in which the porosity (pore morphology or density) shows continuous change along certain direction(s) so as to meet multifunctional requirements. FGPMs have a wide range of potential applications in aerospace, automotive, and biomedical engineering. For example, Deng et al. [4] designed a leaf vein-inspired heat exchanger filled with graded porous structures, exhibiting an improved heat transfer efficiency compared to the uniform counterpart. Zhang et al. [5] proposed a bi-graded foam-filled structure to improve the crashworthiness of vehicles. Perez-Boerema et al. [6] designed a femoral prosthesis characterised by functionally graded porous (FGP) structures as the bone tissue substitute. When such advanced materials are assembled into engineering systems as structural components (e.g., beams, plates, shells, etc.), their mechanical analysis is of great importance for further applications of FGPMs in practical engineering. So far, considerable research efforts have been dedicated to the buckling and free vibration analyses of FGP beams [7,8]. Chen et al. [9,10,11,12] systematically evaluated the buckling and vibration behaviours of FGP beams and indicated that their mechanical performances can be further improved via the symmetric nonuniform porosity distribution. Wattanasakulpong et al. [13,14] conducted the free vibration analysis of FGP beams based on the Chebyshev collocation method. Ebrahimi et al. [15,16] successively investigated effects of rotation and thermal loading on the vibration behaviour of FGP beams. Al Rjoub and Hamad [17] examined the free vibration of FGP beams using the transfer matrix method. Jamshidi and Arghavan [18] studied the optimal distributions of porosity for buckling and free vibration behaviour of FGP beams. Anirudh et al. [19] presented a comprehensive analysis on the buckling, bending, and free vibration of FGP beams by the finite element (FE) approach. Heshmati and Daneshmand [20] assessed the influence of different profile variations of vibrational properties of nonuniform FGP beams. Liu et al. [21] performed a thermal–mechanical coupling buckling analysis of FGP sandwich beams. Askari et al. [22] dealt with the free vibration of FGP beams with piezoelectric layers. Jena et al. [23] analysed the vibration behaviour of FGP beams resting on an elastic foundation. The buckling and free vibration problems of FGP beams were also revisited recently by other researchers based on different beam theories and solution methods [24,25,26,27,28], which are not elaborated here further to avoid repetition.

It is noted that various beam theories are used for buckling and vibration analyses of FGP beams in the abovementioned studies, categorised as the classical beam theory (Euler–Bernoulli beam theory, CBT), first-order shear deformation beam theory (Timoshenko beam theory, FSDBT), and higher-order shear deformation beam theories (HSDBTs), among which the HSDBTs include the widely used third-order shear deformation beam theory (TSDBT) of Reddy, sinusoidal shear deformation beam theory (SSDBT), and exponential shear deformation theory (ESDBT). Table 1 presents the statistics on the beam theories used in the literature. Those theories have proven their accuracy and effectiveness in analysing the mechanical behaviours of isotropic, homogenous, laminated composite and FGM beams. Distinct deformation hypotheses of those theories contribute to the differences of solutions in predicting mechanical behaviours, and the differences become negligible when the beam is slender enough (normally the slenderness ratio is over 10). However, Magnucki and Stasiewicz [29] pointed out earlier that the FGP beam with the maximum/minimum porosity in the middle/outer surfaces will behave like a sandwich beam, and the CBT and FSDBT cannot correctly determine the displacements of the cross-section of the beam. Instead, they proposed a new shear deformation beam theory by making use of the broken-line hypothesis (BSDBT) applied to three-layered structures. Unfortunately, there is an absence of a comprehensive analysis of the accuracy and effectiveness of the BSDBT, and comparative studies between conventional beam theories (CBT, FSDBT, and HSDBT) and the BSDBT in buckling and free vibration analyses of FGP beams have not been reported yet.

Therefore, this paper is devoted to examining the validity and accuracy of four beam theories, namely the CBT, FSDBT, TSDBT, and BSDBT, in analysing the buckling and free vibration behaviours of FGP beams with uniform and nonuniform distributions of porosity. Within the framework of Hamilton’s principle, governing equations of buckling and free vibration are deduced based on each individual beam theory and are all solved by using the generalised differential quadrature (GDQ) method. Comparative studies are conducted through comprehensive numerical examples, with a particular focus on the effects of porosity distribution and coefficient, beam geometry, and boundary conditions on the differences between results of those beam theories. In addition, the accuracy and effectiveness of those beam theories are evaluated by comparing the obtained buckling loads and natural frequencies with FE simulation results. Lastly, the underlying reasons for the discrepancies observed between the results of those theories and simulation are discussed. The present work will serve to better understand the differences and limitations of existing theories for buckling and free vibration analyses of FGP beams and contribute to the establishment of more refined theories for the mechanical analysis of FGP structures.

## 2. Functionally Graded Porous Beam

**Figure 1:**
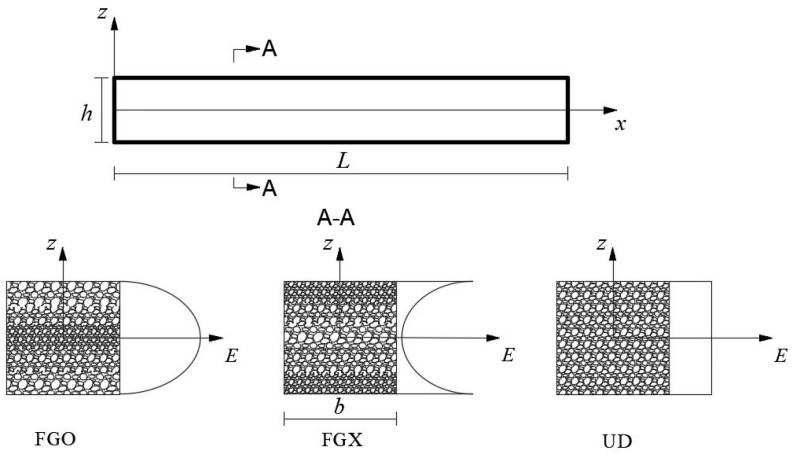
A porous beam with different porosity distributions.

Figure 1 shows a porous beam of length *L* and thickness *h* defined in a rectangular coordinate system. Three porosity gradients that are most commonly investigated in the literature are considered in this study. Among those, patterns FGX/FGO imply that the porosity continuously decreases/increases from the middle to the upper and lower surfaces, while pattern UD means that the porosity is uniformly distributed in the beam. As a result, the elastic moduli either change or remain constant along the thickness direction, which are defined as follows.

(1) $$E(z)=E_{s}[1-e_{0}\varphi (z)],G(z)=G_{s}[1-e_{0}\varphi (z)]$$

in which

(2) $$\varphi (z)=\{\begin{array}{l} \cos\left(\pi z/h\right)\;\;\;\;\;\;\;(\mathrm{FGX})\\ \cos\left(\left|\pi z/h\right|-\pi /2\right)\;\;(\mathrm{FGO})\\ \varphi_{0}\;\;\;\;\;\;\;(\mathrm{UD}) \end{array}$$

*E*_s_ and *G*_s_ are the Young’s modulus and shear modulus of the solid material without pores, respectively; *e*_0_ is the porosity coefficient defined by

(3) $$e_{0}=1-E_{p}/E_{s}=1-G_{p}/G_{s}$$

where *E*_P_ and *G*_P_ are the elastic moduli of the layer with the highest porosity, namely the minimum values of *E*(*z*) and *G*(*z*), respectively. According to the relationship between Young’s modulus and shear modulus, the Poisson’s ratio is given by

(4) ν(z)=E(z)/2G(z)−1

which is apparently a constant for the entire beam.

Several micromechanical models [7] have been developed for the evaluation of mechanical properties of open- and closed-cell porous materials. Among those, the Gibson–Ashby model [30] is the most extensively applied due to its simple form and wide applicability. According to this model, the Young’s modulus of porous materials is related to the mass density as

(5) $$\frac{E(z)}{E_{s}}={\left(\frac{\rho (z)}{\rho_{s}}\right)}^{2}$$

where *ρ*_s_ is the mass density of the solid material. By combining Equations (1) and (5), the mass density of the porous beam can be expressed as

(6) $$\rho (z)=\rho_{s}\sqrt{1-e_{0}\varphi (z)}$$

For direct comparisons, the total mass of porous beams with the same dimension but different porosity gradients should be equal, which requires

(7) $$\int_{-h/2}^{h/2}\rho_{s}\sqrt{1-e_{0}\varphi (z)}dz=\int_{-h/2}^{h/2}\rho_{s}\sqrt{1-e_{0}\varphi_{0}}dz$$

from which the constant coefficient $\varphi_{0}$ for the uniform porosity distribution can be derived as

(8) $$\varphi_{0}=\frac{1}{e_{0}}\left\{1-{\left[\frac{1}{h}\int_{-h/2}^{h/2}\sqrt{1-e_{0}\varphi (z)}dz\right]}^{2}\right\}$$

For the sake of simplicity, the mass density of the porous beam is rewritten in a similar form with the elastic moduli in Equation (1) as

(9) $$\rho (z)=\rho_{s}[1-e_{m}\varphi (z)]$$

in which *e*_m_ can be determined by comparing Equations (6) and (9) as

(10) $$e_{m}=\frac{1}{\varphi (z)}\left[1-\sqrt{1-e_{0}\varphi (z)}\right]$$

## 3. Theoretical Modelling

### 3.1. Beam Theories

**Figure 2:**
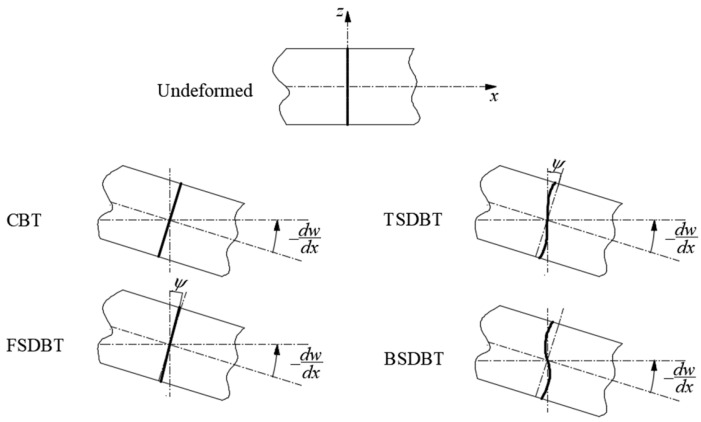
Deformation of the transverse normal according to different beam theories.

In the present study, four beam theories, namely the CBT, FSDBT, TSDBT, and BSDBT, which are most often used in existing studies, are examined for buckling and free vibration analysis of FGP beams. Since different deformation assumptions are applied, the displacement fields of those beam theories differ from each other, as shown in Figure 2.

In the CBT, it is assumed that the transverse normal remains perpendicular to the midplane after deformation, which means that the transverse shear deformation is omitted. In this case, the displacement filed is given by

(11) $$U(x,z,t)=-z\frac{\partial w(x,t)}{\partial x},\;W(x,z,t)=w(x,t)$$

In contrast, the FSDT takes into account the transverse shear deformation by adopting that the transverse normal needs not be perpendicular to the midplane but the cross-section remains a planar surface after deformation. Under this assumption, the displacement field of FSDBT is of the form

(12) U(x,z,t)=zψ(x,t), W(x,z,t)=w(x,t)

However, the assumption on the straightness and normality of a transverse normal after deformation is relaxed in the TSDBT and BSDBT by expanding the displacements as cubic and trigonometric functions, respectively, of the thickness coordinate. Accordingly, the displacement fields are expressed as

(13) $$U(x,z,t)=z\psi (x,t)-\frac{4z^{3}}{3h^{2}}\left(\psi (x,t)+\frac{\partial w(x,t)}{\partial x}\right),\;W(x,z,t)=w(x,t)$$

for the TSDBT and

(14) $$\left\{ \begin{array}{l} U(x,z,t)=-z\frac{\partial w(x,t)}{\partial x}+\frac{h}{\pi }\left[\phi_{1}(x,t)\sin\left(\pi z/h\right)+\phi_{2}(x,t)\sin\left(2\pi z/h\right)\cos^{2}\left(\pi z/h\right)\right]\\ W(x,z,t)=w(x,t) \end{array}\right.$$

for the BSDBT which is established by making use of the broken-line hypothesis applied to three-layered structures [29]. In the above equations, *U* and *W* denote displacements of the beam along the *x* and *z* directions, respectively; *w* represents the transverse displacement component in the midplane (*z* = 0); and *ψ* is the transverse normal rotation about the *x*-axis. *ϕ*_1_ and *ϕ*_2_ are the dimensionless functions of displacements, and *t* is the time. By setting *ϕ*_1_ = *ϕ*_2_ = 0, the BSDBT is reduced to the CBT.

According to the linear strain–displacement relations, the longitudinal tensile and transverse shear strains (*ε_x_*, *γ_xz_*) of different theories are given as

(15) $$\mathrm{CBT}:\;\varepsilon_{x}=-z\frac{\partial^{2}w}{\partial x^{2}},\;\gamma_{xz}=0$$

(16) $$\mathrm{FSDBT}:\;\varepsilon_{x}=z\frac{\partial \psi }{\partial x},\;\gamma_{xz}=\psi +\frac{\partial w}{\partial x}$$

(17) $$\mathrm{TSDBT}:\;\varepsilon_{x}=z\frac{\partial \psi }{\partial x}-c_{1}z^{3}\left(\frac{\partial \psi }{\partial x}+\frac{\partial^{2}w}{\partial x^{2}}\right),\;\gamma_{xz}=\psi +\frac{\partial w}{\partial x}-c_{2}z^{2}\left(\psi +\frac{\partial w}{\partial x}\right)$$

(18) $$\mathrm{BSDBT}:\;\left\{ \begin{array}{l} \varepsilon_{x}=-z\frac{\partial^{2}w}{\partial x^{2}}+\frac{h}{\pi }\left[\frac{\partial \phi_{1}}{\partial x}\sin\left(\pi z/h\right)+\frac{\partial \phi_{2}}{\partial x}\sin\left(2\pi z/h\right)\cos^{2}\left(\pi z/h\right)\right]\\ \gamma_{xz}=\phi_{1}\cos\left(\pi z/h\right)+\phi_{2}\left[\cos\left(2\pi z/h\right)+\cos\left(4\pi z/h\right)\right] \end{array}\right.$$

in which *c*_1_ = 4/3*h*^2^ and *c*_2_ = 4/*h*^2^.

### 3.2. Governing Equations

Consider an FGP beam with the rectangular coordinate system (*x*, *z*) as shown in Figure 1. The governing equations of the beam under a uniform compressive axial load *F* can be formulated by using Hamilton’s principle

(19) $$\delta \int_{t_{1}}^{t_{2}}\left[\prod_{K}-\left(\prod_{S}+\prod_{W}\right)\right]\;dt=0$$

in which the kinetic energy *П_K_*, strain energy *П_S_*, and virtual work *П_W_* performed by the compressive load are given by

(20) $$\prod_{K}=\frac{1}{2}\int_{0}^{L}\int_{-h/2}^{h/2}\rho \left[{\left(\frac{\partial U}{\partial t}\right)}^{2}+{\left(\frac{\partial W}{\partial t}\right)}^{2}\right]dzdx$$

(21) $$\prod_{S}=\frac{1}{2}\int_{0}^{L}\int_{-h/2}^{h/2}\left(E\varepsilon_{x}^{2}+G\gamma_{xz}^{2}\right)dzdx$$

(22) $$\prod_{W}=-\frac{1}{2}{\int_{0}^{L}F\left(\frac{\partial W}{\partial x}\right)}^{2}dx$$

Substituting Equations (20)–(22) into Equation (19) and integrating the parts over the beam thickness, the governing equations for each beam theory are then derived and written in terms of displacement components as

(23) $$D_{11}\frac{\partial^{4}w}{\partial x^{4}}+F\frac{\partial^{2}w}{\partial x^{2}}=-I_{0}\ddot{w}+I_{2}\frac{\partial^{2}\ddot{w}}{\partial x^{2}}$$

for the CBT,

(24) $$\kappa A_{55}\left(\frac{\partial^{2}w}{\partial x^{2}}+\frac{\partial \psi }{\partial x}\right)-F\frac{\partial^{2}w}{\partial x^{2}}=I_{0}\ddot{w}$$

(25) $$D_{11}\frac{\partial^{2}\psi }{\partial x^{2}}-\kappa A_{55}\left(\frac{\partial w}{\partial x}+\psi \right)=I_{2}\ddot{\psi }$$

for the FSDBT, and

(26) $$\begin{array}{l} \left(c_{1}H_{11}-{c_{1}}^{2}J_{11}\right)\frac{\partial^{3}\psi }{\partial x^{3}}-{c_{1}}^{2}J_{11}\frac{\partial^{4}w}{\partial x^{4}}+\left(A_{55}-2c_{2}B_{55}+{c_{2}}^{2}D_{55}\right)\left(\frac{\partial \psi }{\partial x}+\frac{\partial^{2}w}{\partial x^{2}}\right)-F\frac{\partial^{2}w}{\partial x^{2}}\\ =I_{0}\ddot{w}+\left(c_{1}I_{4}-{c_{1}}^{2}I_{6}\right)\frac{\partial \ddot{\psi }}{\partial x}-{c_{1}}^{2}I_{6}\frac{\partial^{2}\ddot{w}}{\partial x^{2}} \end{array}$$

(27) $$\begin{array}{l} \left(D_{11}-2c_{1}H_{11}+{c_{1}}^{2}J_{11}\right)\frac{\partial^{2}\psi }{\partial x^{2}}+\left({c_{1}}^{2}J_{11}-c_{1}H_{11}\right)\frac{\partial^{3}w}{\partial x^{3}}+\left(2c_{2}B_{55}-A_{55}-{c_{2}}^{2}D_{55}\right)\left(\psi +\frac{\partial w}{\partial x}\right)\\ =\left(I_{2}-2c_{1}I_{4}+{c_{1}}^{2}I_{6}\right)\ddot{\psi }+\left({c_{1}}^{2}I_{6}-c_{1}I_{4}\right)\frac{\partial \ddot{w}}{\partial x} \end{array}$$

for the TSDBT. In the above equations, *κ* = 5/6 is the shear correction factor for the FSDBT; the overdot represents the partial derivative with respect to time; and the stiffness elements *A_ij_*, *B_ij_*, *D_ij_*, *F_ij_*, *H_ij_*, and *J_ij_* and inertia-related term *I_i_* are defined as

(28) $$\left(A_{11},B_{11},D_{11},F_{11},H_{11},J_{11}\right)=\int_{-h/2}^{h/2}E(z)(1,z,z^{2},z^{3},z^{4},z^{6})\;dz$$

(29) $$(A_{55},B_{55},D_{55})=\int_{-h/2}^{h/2}G(z)(1,z^{2},z^{4})\;dz$$

(30) $$I_{i}=\int_{-h/2}^{h/2}\rho (z){\left(z\right)}^{i}dz,\;\;\;\;\;\;(i=0,1,2,\cdots ,6)$$

For the BSDBT, the governing equations are expressed as

(31) $$E_{s}h^{3}\left(C_{1}\frac{\partial^{4}w}{\partial x^{4}}-C_{2}\frac{\partial^{3}\phi_{1}}{\partial x^{3}}-C_{3}\frac{\partial^{3}\phi_{2}}{\partial x^{3}}\right)+F\frac{\partial^{2}w}{\partial x^{2}}=\rho_{s}h^{3}\left(B_{1}\frac{\partial^{2}\ddot{w}}{\partial x^{2}}-\frac{I_{0}}{h^{3}}\ddot{w}-B_{2}\frac{\partial {\ddot{\phi }}_{1}}{\partial x}-B_{3}\frac{\partial {\ddot{\phi }}_{2}}{\partial x}\right)$$

(32) $$E_{s}\left(C_{2}\frac{\partial^{3}w}{\partial x^{3}}-C_{4}\frac{\partial^{2}\phi_{1}}{\partial x^{2}}-C_{5}\frac{\partial^{2}\phi_{2}}{\partial x^{2}}\right)+\frac{E_{s}}{2h^{2}\left(1+v\right)}\left(C_{7}\phi_{1}+C_{8}\phi_{2}\right)=\rho_{s}\left(B_{2}\frac{\partial \ddot{w}}{\partial x}-B_{4}{\ddot{\phi }}_{1}-B_{5}{\ddot{\phi }}_{2}\right)$$

(33) $$E_{s}\left(C_{3}\frac{\partial^{3}w}{\partial x^{3}}-C_{5}\frac{\partial^{2}\phi_{1}}{\partial x^{2}}-C_{6}\frac{\partial^{2}\phi_{2}}{\partial x^{2}}\right)+\frac{E_{s}}{2h^{2}\left(1+v\right)}\left(C_{8}\phi_{1}+C_{9}\phi_{2}\right)=\rho_{s}\left(B_{3}\frac{\partial \ddot{w}}{\partial x}-B_{5}{\ddot{\phi }}_{1}-B_{6}{\ddot{\phi }}_{2}\right)$$

in which

(34) $$B_{i}=\frac{1}{h}\int_{-h/2}^{h/2}\left[1-e_{m}\varphi (z)\right]\xi_{i}(z)dz,\;C_{i}=\frac{1}{h}\int_{-h/2}^{h/2}\left[1-e_{0}\varphi (z)\right]\xi_{i}(z)dz\;\;(i=0,1,2,\cdots ,9)$$

By setting η=z/h, *ξ_i_* in Equation (34) are given by

(35) $$\xi_{1}=\eta^{2},\;\xi_{2}=\eta \sin\left(\pi \eta \right),\;\xi_{3}=\frac{\eta }{\pi }\sin\left(2\pi \eta \right)\cos^{2}\left(\pi \eta \right),\;\xi_{4}=\frac{1}{\pi^{2}}\sin^{2}\left(\pi \eta \right)\;\xi_{5}=\frac{1}{\pi^{2}}\sin\left(\pi \eta \right)\cos^{2}\left(\pi \eta \right)\sin\left(2\pi \eta \right),\;\xi_{6}=\frac{1}{\pi^{2}}\sin^{2}\left(2\pi \eta \right)\cos^{4}\left(\pi \eta \right),\;\xi_{7}=\cos^{2}\left(\pi \eta \right)\;\xi_{8}=\cos\left(\pi \eta \right)\left[\cos\left(2\pi \eta \right)+\cos\left(4\pi \eta \right)\right],\;\xi_{9}={\left[\cos\left(2\pi \eta \right)+\cos\left(4\pi \eta \right)\right]}^{2}$$

Two different end supports, namely hinged (H) and clamped (C), are considered in this study. The corresponding boundary conditions in terms of displacements are given in Table 2.

### 3.3. Solution Procedure

Since it is difficult to obtain closed-form solutions, a numerical method such as the GDQ method [31,32] is adopted to solve the abovementioned differential governing equations. According to this method, the displacement components *w*, *ψ*, *ϕ*_1_, and *ϕ*_2_ and their *m*^th^ partial derivatives with respect to *x* are approximated by

(36) $$\left\{ \begin{array}{l} {\left\{w,\psi ,\phi_{1},\phi_{2}\right\}|}_{x=x_{i}}=\sum_{j=1}^{N}l_{j}(x_{i})\left\{w_{j},\psi_{j},\;\phi_{1j},\;\phi_{2j}\right\}\\ {\frac{\partial^{m}}{\partial x^{m}}\left\{w,\;\psi ,\phi ,\phi_{1},\;\phi_{2}\right\}|}_{x=x_{i}}=\sum_{j=1}^{N}G_{ij}^{\left(m\right)}\left\{w_{j},\;\psi_{j},\phi_{1j},\;\phi_{2j}\right\} \end{array}\right.$$

where {*w_j_*, *ψ_j_*, *ϕ*_1*j*_, *ϕ*_2*j*_} are the values of {*w*, *ψ*, *ϕ*_1_, *ϕ*_2_} at *x* = *x_j_*; *l_j_*(*x*) are the Lagrange interpolation polynomials; and $G_{ij}^{\left(m\right)}$ are the weighting coefficients of the *m*^th^ derivatives and are determined by the recursive formula [33]. *N* is the total number of grid points which are spaced along the *x*-axis according to the following pattern:

(37) $$x_{i}=\frac{1}{2}\left[1-\cos\frac{\pi (i-1)}{N-1}]\;(i=1,2,\ldots ,N\right)$$

By applying the relationship in (36), the differential governing equations of each beam theory can be discretised as

(38) $$D_{11}\sum_{j=1}^{N}G_{ij}^{\left(4\right)}w_{j}+F\sum_{j=1}^{N}G_{ij}^{\left(2\right)}w_{j}=-I_{0}{\ddot{w}}_{i}+I_{2}\sum_{j=1}^{N}G_{ij}^{\left(2\right)}{\ddot{w}}_{j}$$

for the CBT,

(39) $$A_{55}(\sum_{j=1}^{N}G_{ij}^{\left(2\right)}w_{j}+\sum_{j=1}^{N}G_{ij}^{\left(1\right)}\psi_{j})-F\sum_{j=1}^{N}G_{ij}^{\left(2\right)}w_{j}=I_{0}{\ddot{w}}_{i}$$

(40) $$D_{11}\sum_{j=1}^{N}G_{ij}^{\left(2\right)}\psi_{j}-A_{55}(\sum_{j=1}^{N}G_{ij}^{\left(1\right)}w_{j}+\psi_{i})=I_{2}{\ddot{\psi }}_{i}$$

for the FSDBT,

(41) $$\begin{array}{l} \left(c_{1}H_{11}-{c_{1}}^{2}J_{11}\right)\sum_{j=1}^{N}G_{ij}^{\left(3\right)}\psi_{j}-{c_{1}}^{2}J_{11}\sum_{j=1}^{N}G_{ij}^{\left(4\right)}w_{j}+\left(A_{55}-2c_{2}B_{55}+{c_{2}}^{2}D_{55}\right)\left(\sum_{j=1}^{N}G_{ij}^{\left(1\right)}\psi_{j}+\sum_{j=1}^{N}G_{ij}^{\left(2\right)}w_{j}\right)\\ -F\sum_{j=1}^{N}G_{ij}^{\left(2\right)}w_{j}=I_{0}\ddot{w}+\left(c_{1}I_{4}-{c_{1}}^{2}I_{6}\right)\sum_{j=1}^{N}G_{ij}^{\left(1\right)}{\ddot{\psi }}_{j}-{c_{1}}^{2}I_{6}\sum_{j=1}^{N}G_{ij}^{\left(2\right)}{\ddot{w}}_{j} \end{array}$$

(42) $$\begin{array}{l} \left(D_{11}-2c_{1}H_{11}+{c_{1}}^{2}J_{11}\right)\sum_{j=1}^{N}G_{ij}^{\left(2\right)}\psi_{j}+\left({c_{1}}^{2}J_{11}-c_{1}H_{11}\right)\sum_{j=1}^{N}G_{ij}^{\left(3\right)}w_{j}\\ +\left(2c_{2}B_{55}-A_{55}-{c_{2}}^{2}D_{55}\right)\left(\psi_{i}+\sum_{j=1}^{N}G_{ij}^{\left(1\right)}w_{j}\right)=\left(I_{2}-2c_{1}I_{4}+{c_{1}}^{2}I_{6}\right){\ddot{\psi }}_{i}+\left({c_{1}}^{2}I_{6}-c_{1}I_{4}\right)\sum_{j=1}^{N}G_{ij}^{\left(1\right)}{\ddot{w}}_{j} \end{array}$$

for the TSDBT, and

(43) $$\begin{array}{l} E_{s}h^{3}\left(C_{1}\sum_{j=1}^{N}G_{ij}^{\left(4\right)}w_{j}-C_{2}\sum_{j=1}^{N}G_{ij}^{\left(3\right)}\phi_{1j}-C_{3}\sum_{j=1}^{N}G_{ij}^{\left(3\right)}\phi_{2j}\right)+F\sum_{j=1}^{N}G_{ij}^{\left(2\right)}w_{j}\\ =\rho_{s}h^{3}\left(B_{1}\sum_{j=1}^{N}G_{ij}^{\left(2\right)}{\ddot{w}}_{j}-\frac{I_{0}}{h^{3}}{\ddot{w}}_{i}-B_{2}\sum_{j=1}^{N}G_{ij}^{\left(1\right)}{\ddot{\phi }}_{1j}-B_{3}\sum_{j=1}^{N}G_{ij}^{\left(1\right)}{\ddot{\phi }}_{2j}\right) \end{array}$$

(44) $$\begin{array}{l} E_{s}\left(C_{2}\sum_{j=1}^{N}G_{ij}^{\left(3\right)}w_{j}-C_{4}\sum_{j=1}^{N}{Κ}_{ij}^{\left(2\right)}\phi_{1j}-C_{5}\sum_{j=1}^{N}G_{ij}^{\left(2\right)}\phi_{2j}\right)+\frac{E_{s}}{2h^{2}\left(1+v\right)}\left(C_{7}\phi_{1i}+C_{8}\phi_{2i}\right)\\ =\rho_{s}\left(B_{2}\sum_{j=1}^{N}G_{ij}^{\left(1\right)}{\ddot{w}}_{j}-B_{4}{\ddot{\phi }}_{1i}-B_{5}{\ddot{\phi }}_{2i}\right) \end{array}$$

(45) $$\begin{array}{l} E_{s}\left(C_{3}\sum_{j=1}^{N}G_{ij}^{\left(3\right)}w_{j}-C_{5}\sum_{j=1}^{N}G_{ij}^{\left(2\right)}\phi_{1j}-C_{6}\sum_{j=1}^{N}G_{ij}^{\left(2\right)}\phi_{2j}\right)+\frac{E_{s}}{2h^{2}\left(1+v\right)}\left(C_{8}\phi_{1i}+C_{9}\phi_{2i}\right)\\ =\rho_{s}\left(B_{3}\sum_{j=1}^{N}G_{ij}^{\left(1\right)}{\ddot{w}}_{j}-B_{5}{\ddot{\phi }}_{1i}-B_{6}{\ddot{\phi }}_{2i}\right) \end{array}$$

for the BSDBT. The boundary conditions in Table 2 can be handled in the same way, and their discretised forms are given in Table 3.

Substitution of the boundary conditions in Table 3 into the discretised governing equations of each theory leads to an algebraic system that governs the buckling and free vibration of the beam and can be written in a matrix form as

(46) $$M\ddot{d}+\left(K-FK_{F}\right)d=0$$

in which **M** is the mass matrix; **K** and **K**_F_ are the stiffness matrix and geometric stiffness matrix, respectively; and the unknown displacement vector **d** is composed of *w_j_*, *ψ_j,_* or *ϕ_kj_* (*k* = 1, 2; *j* = 1, 2, …, *N*).

For the buckling analysis, the inertia terms are omitted and Equation (46) reduces to an eigenvalue equation as

(47) $$\left(K-FK_{F}\right)d=0$$

from which the critical buckling load can be obtained as the lowest positive eigenvalue.

For the free vibration analysis, the axial load *F* is absent and the displacement vector **d** is expressed as **d** = d*sin(*Ωt*). Equation (46) is then rewritten as

(48) $$\left(K-\Omega^{2}M\right)d=0$$

where *Ω* is the natural frequency and can be determined through a standard eigenvalue algorithm.

## 4. Finite Element Simulation

**Figure 3:**
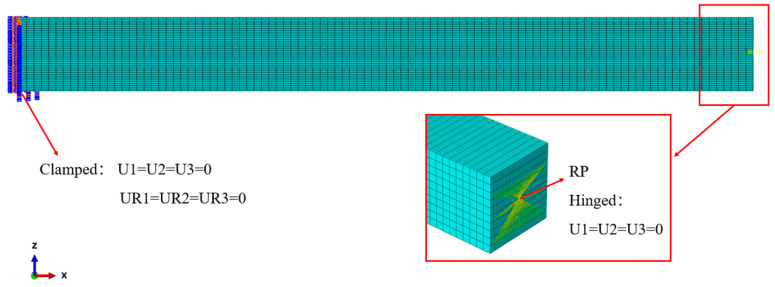
FE model of the FG porous beam in ABAQUS.

FE simulation is a well-accepted tool for structural analysis, and the simulation results are used in this paper for direct comparison with those of different beam theories. To begin with, the FE model of the FG porous beam is created in the commercial software ABAQUS 2016 and meshed with eight-node reduced integration hexahedral elements (C3D8R) of controlled size, as shown in Figure 3. Due to the graded distribution of porosity, the material properties of the FG porous beam change continuously along the thickness direction, which is difficult to implement in the software. As an alternative, the continuous variation in material properties is approximated in a stepwise manner. To this end, the FG porous beam model is divided into a number of layers of equal thickness via the “*offset faces*” option, and the material properties are constant in each individual layer but change from layer to layer across the thickness. For the buckling analysis, an axial compressive load is applied at the reference point (RP) that couples with the right end of the beam. The step procedure named “*Linear perturbation/Buckle*” is selected to obtain the critical buckling load. For the free vibration analysis, the “*Linear perturbation/Frequency*” procedure is used to determine the natural frequency. Various numbers of layers were examined and it is established that a total number of 40 layers will suffice in simulating the FGP beam with continuous material distributions and furnishing converged simulation results.

## 5. Results and Discussion

In this section, we examine the validity and accuracy of the four beam theories in predicting the critical buckling load and fundamental frequency of FG porous beams by comparing them with their counterparts obtained from FE simulations. It is assumed that the porous beams of thickness *h* = 0.1 m are made from steel with material properties as follows: *E*_s_ = 200 GPa, vs. = 0.3, and *ρ*_s_ = 7850 kg/m^3^. Unless otherwise stated, the critical buckling load and fundamental frequency results are given in the dimensionless form as

(49) $$f_{\mathrm{cr}}=\frac{100F_{\mathrm{cr}}}{hE_{s}},\;\omega =10\Omega h\sqrt{\frac{\rho_{s}}{E_{s}}}$$

### 5.1. Convergence and Validation

Convergence analysis is first conducted, and the dimensionless buckling and free vibration results of the FG porous beams with different numbers of grid points are tabulated and compared in Table 4. It is seen that the present solutions are convergent for all beam theories when the total number of grid points is increased to *N* = 27. Hence, *N* = 27 is used in the subsequent calculations. Following that, validation analysis is performed, and the dimensionless critical buckling loads and fundamental frequencies of FG porous beams with different porosity gradients are calculated and compared in Table 5 with the FE simulation results, as well as those in the literature [24]. As can be observed, the present results agree well with the simulation and existing ones.

Nevertheless, Table 4 and Table 5 show that there exist differences between the results of the four beam theories, and the differences become much more significant for FGX porous beams with a high porosity (*e*_0_ = 0.99). To understand this, the difference and accuracy of those beam theories in predicting the critical buckling load and fundamental frequency of FG porous beams are comprehensively examined through various numerical examples in the following sections.

### 5.2. Buckling Analysis

We first compare the buckling results predicted by the theories with those of FE simulation in Table 6 for the FG porous beams with different boundary conditions and porosity coefficients. *e*_0_ = 0 represents that the beam is homogeneous without pores. As expected, the porosity gradient FGX with fewer pores near the surfaces provides the highest stiffness and possesses the greatest critical buckling loads, followed by UD and FGO. Compared to other theories, the CBT always gives the highest predictions due to the fact that the transverse shear deformation is neglected in this theory. In contrast, the FSDBT, TSDBT, and BSDBT furnish very close critical buckling loads to the simulation results for the FGO and UD porous beams, regardless of the porosity value and boundary conditions. Thus, the following discussion centres on the FGX porous beams. From the results of FGX porous beams in Table 6, it is clear that the BSDBT predictions are closer to the simulation ones than other theories, and the difference between those theories and simulation results changes with respect to the porosity coefficient and boundary conditions.

**Figure 4:**
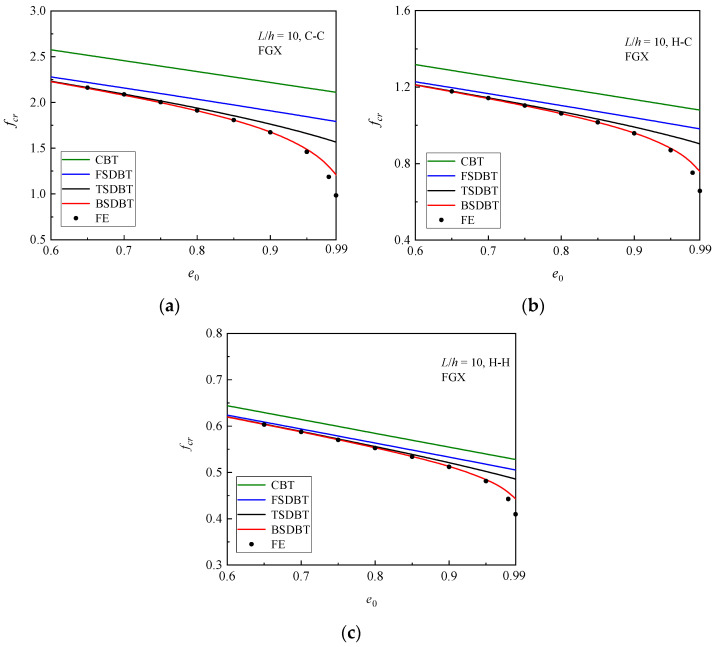
Dimensionless critical buckling load versus porosity coefficient curves of FGX porous beams: (**a**) C-C; (**b**) H-C; (**c**) H-H.

For a better visualisation, the variations in the critical buckling load with respect to the porosity coefficient are plotted in Figure 4 for the FGX porous beams with different boundary conditions. The figure shows that the critical buckling load decreases but the difference between the results of theories and simulations rises as the porosity coefficient increases, and these effects become much more pronounced when the porosity coefficient exceeds 0.9. Nonetheless, the BSDBT predictions are always the closest to the simulation results, followed by those of the TSDBT, FSDBT, and CBT in order.

**Figure 5:**
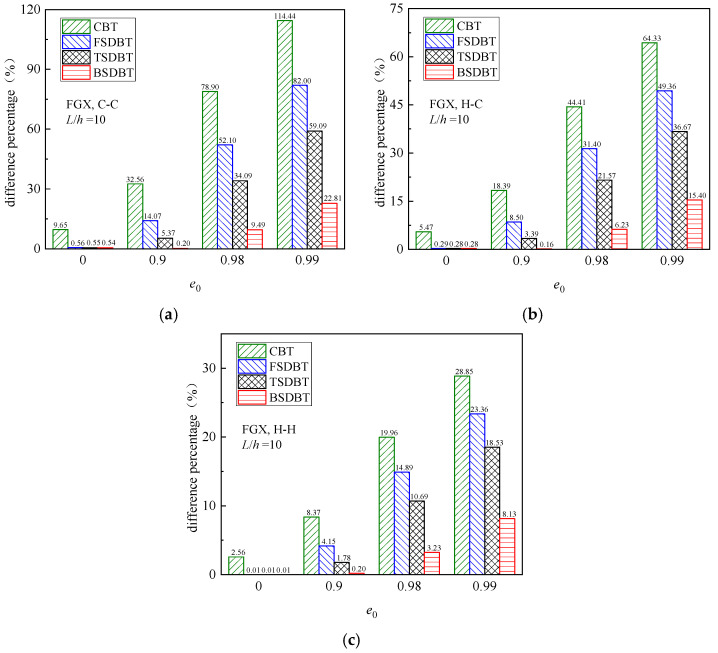
Effect of porosity coefficient on the difference between the buckling results of various theories and FE simulations: (**a**) C-C; (**b**) H-C; (**c**) H-H.

To evaluate the accuracy of those theories at higher porosity values (*e*_0_ = 0.9, 0.98, and 0.99), the difference between the results obtained from theories and simulation are calculated by

(50) $$difference=\frac{\left|f_{cr}(\mathrm{FE})-f_{cr}(\mathrm{Theory})\right|}{f_{cr}(\mathrm{FE})}\times 100\%$$

and compared in Figure 5, where results for *e*_0_ = 0 are also provided as a reference. It is concluded from the figure that for the homogeneous beam (*e*_0_ = 0), all the shear deformation beam theories provide very close predictions to the simulation results. However, for the high porosity values (*e*_0_ ≥ 0.9), the difference between the results of theories and simulations significantly increases, and this effect turns out to be less noticeable for the soft (hinged) end supports. It should be noted that when the porosity coefficient *e*_0_ is increased to 0.98, only the BSDBT gives close predictions to the simulation results, with a difference less than 10%. Unfortunately, when *e*_0_ = 0.99, the differences go up to 22.8% and 15.4% for the C-C and H-C end supports, respectively.

**Figure 6:**
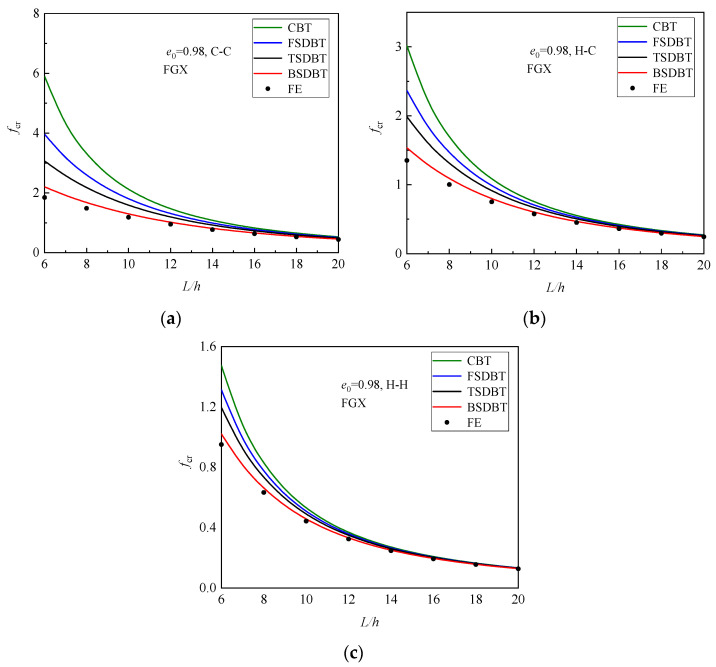
Dimensionless critical buckling load versus slenderness ratio curves of FGX porous beams: (**a**) C-C; (**b**) H-C; (**c**) H-H.

**Figure 7:**
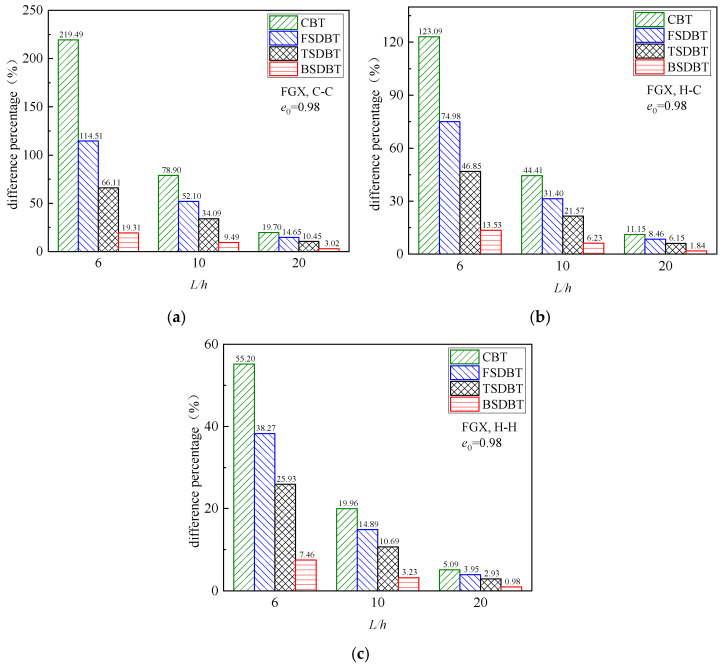
Effect of slenderness ratio on the difference between the buckling results of various theories and simulations: (**a**) C-C; (**b**) H-C; (**c**) H-H.

Figure 6 displays the variation in critical buckling load with the slenderness ratio for the FGX porous beams with different boundary conditions. The critical buckling load and the difference between those beam theories declines as the slenderness ratio increases because a higher slenderness ratio leads to a lower stiffness and a weaker transverse shear effect. The differences between the results of various theories and the simulation at *L*/*h* = 6, 10, and 20 are further plotted and compared in Figure 7. It is seen that only the BSDBT provides very close predictions to the simulation ones with differences less than 10% when the slenderness ratio is increased to 10. The differences are further reduced to 3.02%, 1.84%, and 0.98% for the C-C, H-C, and H-H end supports in order when *L*/*h* = 20.

### 5.3. Free Vibration Analysis

We next investigate the accuracy and difference of those beam theories in predicting the fundamental frequencies of FG porous beams. Table 7 compares the fundamental frequency results of different beam theories and FE simulations for FG porous beams with different boundary conditions and porosity coefficients. Similar to the observations in buckling analysis, the FGX porous beam has the highest fundamental frequency and all shear deformation theories give very close predictions to simulation results for UD and FGO porous beams. Again, there exist certain differences between the results of theories and simulations for FGX porous beams, and the differences turn out to be extremely large at *e*_0_ = 0.99.

**Figure 8:**
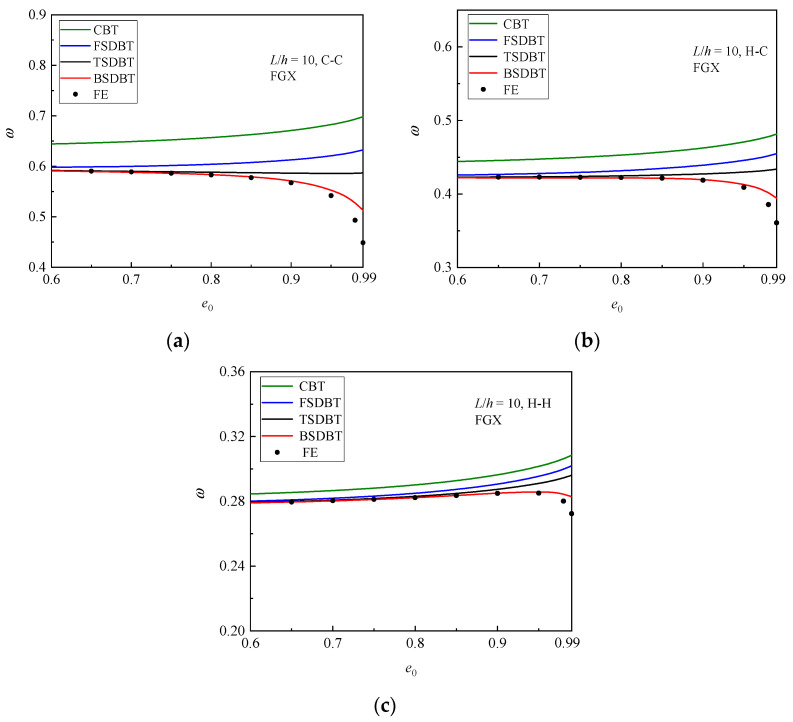
Dimensionless fundamental frequency versus porosity coefficient curves of FGX porous beams: (**a**) C-C; (**b**) H-C; (**c**) H-H.

**Figure 9:**
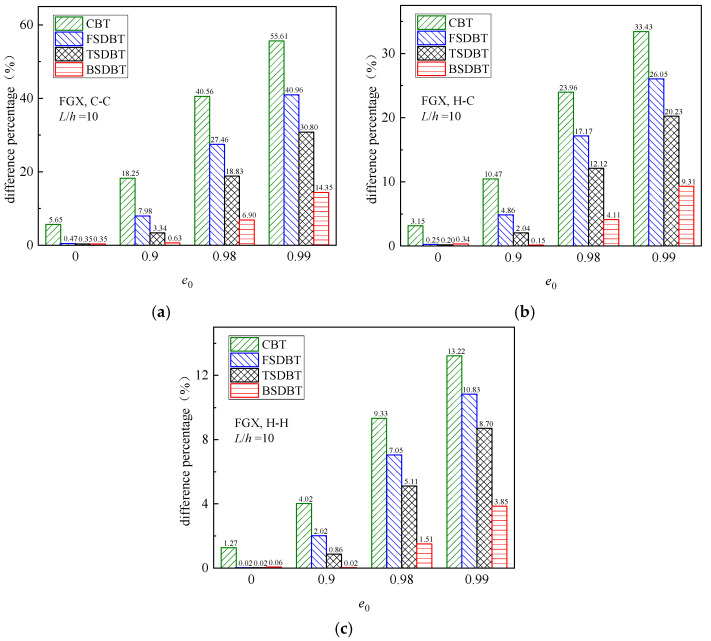
Effect of porosity coefficient on the difference between the frequency results of various theories and FE simulations: (**a**) C-C; (**b**) H-C; (**c**) H-H.

To further illustrate the influence of the porosity coefficient, Figure 8 depicts the fundamental frequency versus porosity coefficient curves of FGX porous beams with different boundary conditions. According to the CBT, FSDBT, and TSDBT curves, the fundamental frequency slightly increases as the porosity coefficient rises. However, both the BSDBT and simulation results show that the frequency decreases with the porosity coefficient increasing, and this effect becomes much more noticeable when the porosity value *e*_0_ increases from 0.9 to 0.99. The figure also shows that all the shear deformation theories give very close predictions to simulation results when *e*_0_ < 0.9, but there are distinct differences between the results obtained from the theories and simulation when *e*_0_ > 0.9. The differences at high porosity values (*e*_0_ = 0.9, 0.98, and 0.99) are calculated and compared in Figure 9. Again, the difference is observably elevated when the porosity coefficient is increased to 0.99 and the end supports become more rigid. Nonetheless, the BSDBT always provides the closest predictions to simulation results, with the maximum differences of 6.90% and 14.35% at *e*_0_ = 0.98 and 0.99, respectively.

**Figure 10:**
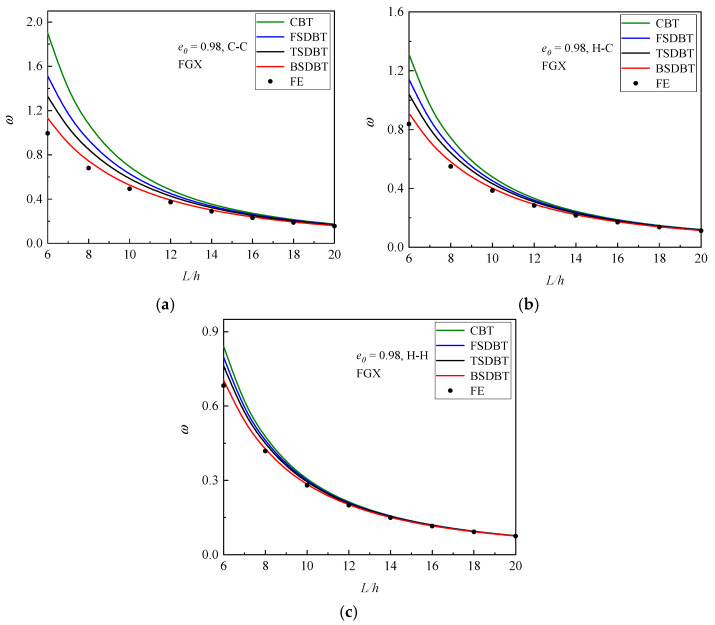
Dimensionless fundamental frequency versus slenderness ratio curves of FGX porous beams: (**a**) C-C; (**b**) H-C; (**c**) H-H.

**Figure 11:**
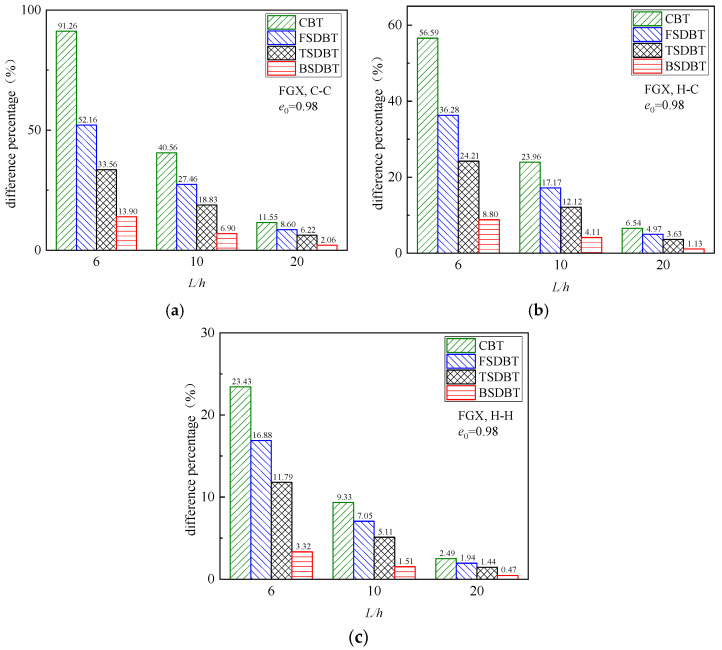
Effect of slenderness ratio on the difference between the frequency results of various theories and simulations: (**a**) C-C; (**b**) H-C; (**c**) H-H.

Figure 10 shows the variation in the fundamental frequency with the slenderness ratio for the FGX porous beams with different boundary conditions. As observed in the buckling analysis, a higher slenderness ratio leads to a lower stiffness and thus results in smaller fundamental frequency and difference between the theories and simulation. For a better illustration, the differences between the results of various theories and simulation at *L*/*h* = 6, 10, and 20 are computed and compared in Figure 11. It is seen that for a thick FGX porous beam (*L*/*h* = 6), only the BSDBT furnishes reasonable predictions with the differences of 13.9%, 8.8%, and 3.32% for the C-C, H-C, and H-H end supports, respectively. As the slenderness ratio is increased to 20, all the shear deformation theories give close predictions with the maximum difference of 8.60%. It is also found that for the same beam, the difference of the frequency results is smaller than that of buckling results.

### 5.4. Analysis of Reasons for the Differences

**Figure 12:**
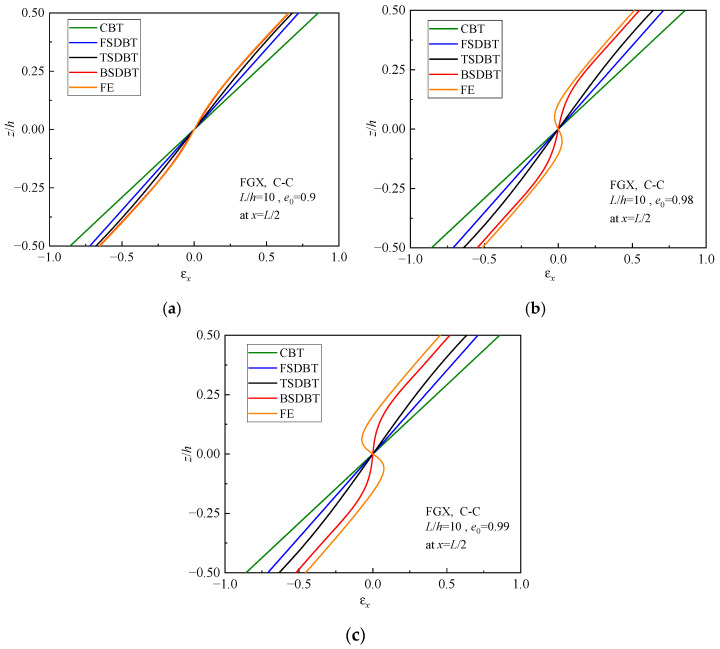
Normal strain distributions in the cross-section of FGX porous beams: (**a**) *e*_0_ = 0.9; (**b**) *e*_0_ = 0.98; (**c**) *e*_0_ = 0.99.

**Figure 13:**
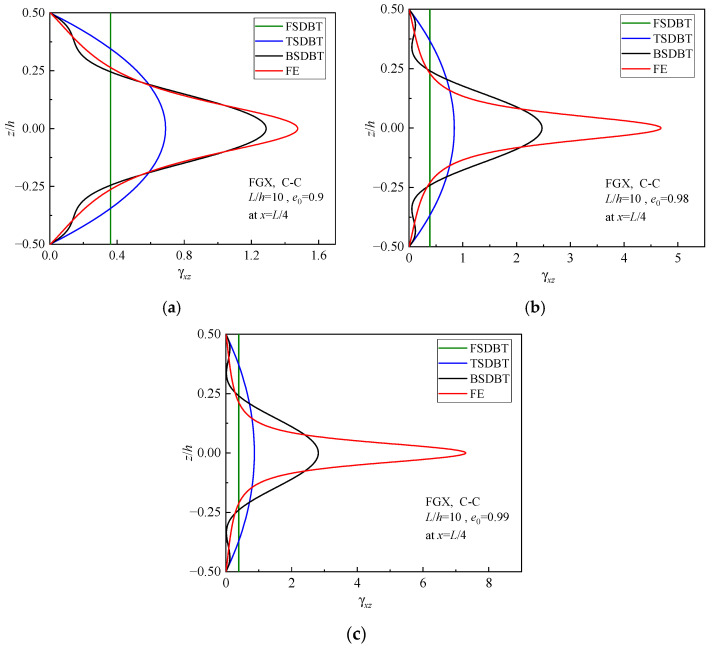
Shear strain distributions in the cross-section of FGX porous beams: (**a**) *e*_0_ = 0.9; (**b**) *e*_0_ = 0.98; (**c**) *e*_0_ = 0.99.

The discrepancies between various theoretical predictions and simulation results can be attributed to different deformation hypotheses adopted in the beam theories, as mentioned in Section 3.1. The distinct displacement functions result in different normal and shear strain distributions in the cross-section of the beam, and they consequently give rise to the deviation of predictions. Figure 12 and Figure 13 display the normal and shear strain curves, respectively, of FGX porous beams with different porosity coefficients. It is seen that the differences between the strain curves of various theories and simulations become greater as the porosity increases. Particularly, when the porosity is increased to 0.99, the moduli at the top and bottom surfaces of the FGX beam are 100 times those at the midplane. In this case, the FGX beam will behave like a three-layered beam, and the beam theories cannot correctly determine the shear strain distribution in the cross-section. This is why all the theories lose their accuracy of prediction at *e*_0_ = 0.99. Nonetheless, the strain curves of BSDBT are always closest to the simulation ones, which explains why the BSDBT solutions are invariably the closest to simulation ones.

## 6. Conclusions

In this paper, we have examined the validity and accuracy of various beam theories (CBT, FSDBT, BSDBT, and BSDBT) in predicting the buckling loads and fundamental frequencies of FG porous beams on the basis of the FE simulation results. Tabular and graphical results are presented to evaluate the influences of the porosity distribution and coefficient, slenderness ratio, and boundary conditions on differences between various theoretical predictions and simulation results. It is found that all beam theories give very close predictions for FGO and UD porous beams but show distinct deviations for FGX porous beams with high porosity. The differences between various theoretical and simulation results significantly increase as the porosity increases beyond 0.9 and the end supports become more rigid, but they decline as the slenderness ratio increases. The underlying reasons for the discrepancies observed between the theoretical predictions and simulation results have been analysed. Among those beam theories, the BSDBT always furnishes the closest predictions to simulation results because it is able to more adequately capture the deformations of the cross-section of FGX porous beams. Nonetheless, the BSDBT will also lose its accuracy when the FGX beam is fully clamped and the porosity is increased to 0.99. Those research findings are instrumental in developing enhanced theoretical models for the mechanical analysis of FGP structures.

## Figures and Tables

**Table 1:** Statistics on the beam theories used in the literature (the blank cell indicates a duplication of the content above).

Theories		Years	References	Mechanical Behaviours
CBT		2016	[16]	vibration
		2017	[17]	
		2021	[22]	
FSDBT		2015	[9]	buckling, bending
			[13]	vibration
			[15]	
		2016	[10]	
		2017	[11] [17]	buckling, vibration vibration
		2018	[18]	buckling, vibration
		2019	[20]	vibration
		2021	[22]	
		2022 2023	[12] [27]	buckling, vibration
HSDBT	TSDBT	2018 2021	[14] [22]	vibration
		2022	[24]	buckling, bending, vibration
		2023	[25]	
		2024	[26] [27]	buckling, vibration vibration
	SSDBT	2019	[19]	buckling, bending, vibration
			[21]	buckling
		2021	[22]	vibration
		2021	[23]	
	ESDBT	2021	[22]	vibration

**Table 2:** Boundary conditions for different beam theories.

Beam Theory	End Support	Boundary Conditions
CBT	Hinged	w=0 $,\;\frac{\partial^{2}w}{\partial x^{2}}=0$
	Clamped	w=0 $,\;\frac{\partial w}{\partial x}=0$
FSDBT	Hinged	w=0 $,\;\frac{\partial \psi }{\partial x}=0$
	Clamped	w=0 , ψ=0
TSDBT	Hinged	w=0 $,\;\frac{\partial^{2}w}{\partial x^{2}}=0$ $,\;\frac{\partial \psi }{\partial x}=0$
	Clamped	w=0 $,\;\frac{\partial w}{\partial x}=0$ , ψ=0
BSDBT	Hinged	w=0$,\;\frac{\partial^{2}w}{\partial x^{2}}=0$$,\;\frac{\partial \phi_{k}}{\partial x}=0$ (*k* = 1, 2)
	Clamped	w=0$,\;\frac{\partial w}{\partial x}=0$$,\;\phi_{k}=0$ (*k* = 1, 2)

**Table 3:** Discretised boundary conditions for different beam theories.

Beam Theory	End Support	Boundary Conditions
CBT	Hinged	$w_{1}=0$$,\;\sum_{j=1}^{N}G_{2j}^{\left(2\right)}w_{j}=0$ at *x* = 0 $w_{N}=0$$,\;\sum_{j=1}^{N}G_{(N-1)j}^{\left(2\right)}w_{j}=0$ at *x* = *L*
	Clamped	$w_{1}=0$$,\;\sum_{j=1}^{N}G_{2j}^{\left(1\right)}w_{j}=0$ at *x* = 0 $w_{N}=0$$,\;\sum_{j=1}^{N}G_{(N-1)j}^{\left(1\right)}w_{j}=0$ at *x* = *L*
FSDBT	Hinged	$w_{1}=0$$,\;\sum_{j=1}^{N}G_{1j}^{\left(1\right)}\psi_{j}=0$ at *x* = 0 $w_{N}=0$$,\;\sum_{j=1}^{N}G_{Nj}^{\left(1\right)}\psi_{j}=0$ at *x* = *L*
	Clamped	$w_{1}=0$$,\;\psi_{1}=0$ at *x* = 0 $w_{N}=0$$,\;\psi_{N}=0$ at *x* = *L*
TSDBT	Hinged	$w_{1}=0$$,\;\sum_{j=1}^{N}G_{2j}^{\left(2\right)}w_{j}=0$$,\;\sum_{j=1}^{N}G_{1j}^{\left(1\right)}\psi_{j}=0$ at *x* = 0 $w_{N}=0$$,\;\sum_{j=1}^{N}G_{(N-1)j}^{\left(2\right)}w_{j}=0$$,\;\sum_{j=1}^{N}G_{Nj}^{\left(1\right)}\psi_{j}=0$ at *x* = *L*
	Clamped	$w_{1}=0$$,\;\psi_{1}=0$$,\;\sum_{j=1}^{N}G_{1j}^{\left(1\right)}w_{j}=0$ at *x* = 0 $w_{N}=0$$,\;\psi_{N}=0$$,\;\sum_{j=1}^{N}G_{Nj}^{\left(1\right)}w_{j}=0$ at *x* = *L*
BSDBT	Hinged	$w_{1}=0$$,\;\sum_{j=1}^{N}G_{2j}^{\left(2\right)}w_{j}=0$$,\;\sum_{j=1}^{N}G_{1j}^{\left(1\right)}\phi_{kj}=0$ at *x* = 0 $w_{N}=0$$,\;\sum_{j=1}^{N}G_{(N-1)j}^{\left(2\right)}w_{j}=0$$,\;\sum_{j=1}^{N}G_{Nj}^{\left(1\right)}\phi_{kj}=0$ at *x* = *L* (*k* = 1, 2)
	Clamped	$w_{1}=0$$,\;\sum_{j=1}^{N}G_{2j}^{\left(1\right)}w_{j}=0$$,\;\phi_{k1}=0$ at *x* = 0 $w_{N}=0$$,\;\sum_{j=1}^{N}G_{(N-1)j}^{\left(1\right)}w_{j}=0$$,\;\phi_{kN}=0$ at *x* = *L* (*k* = 1, 2)

**Table 4:** Dimensionless critical buckling loads and fundamental frequencies of the FGX porous beam varying with the total number of grid points (C-C, *L*/*h* = 10, *e*_0_ = 0.99).

*N*	*f_cr_*				*ω*			
	CBT	FSDBT	TSDBT	BSDBT	CBT	FSDBT	TSDBT	BSDBT
7	2.2697	1.7505	1.5349	1.0178	0.7041	0.6298	0.6242	0.1370
11	2.1117	1.7922	1.5666	0.0791	0.6982	0.6325	0.5932	0.4348
15	2.1116	1.7922	1.5666	0.2115	0.6982	0.6325	0.5886	0.5042
19	2.1116	1.7922	1.5666	0.4387	0.6982	0.6325	0.5873	0.5115
23	2.1116	1.7922	1.5666	0.7764	0.6982	0.6325	0.5870	0.5127
27	2.1116	1.7922	1.5666	1.2093	0.6982	0.6325	0.5869	0.5130
31	2.1116	1.7922	1.5666	1.2093	0.6982	0.6325	0.5869	0.5130

**Table 5:** Comparison of dimensionless critical buckling loads and fundamental frequencies for the FG porous beams with different porosity distributions (C-C, *L*/*h* = 20, *e*_0_ = 0.8).

Source	$f_{\mathrm{cr}}=2(1-\nu^{2})F_{\mathrm{cr}}/hE_{s}$			$\omega =\Omega L\sqrt{\rho_{s}(1-\nu^{2})/E_{s}}$		
	UD	FGX	FGO	UD	FGX	FGO
CBT	69.091	116.887	48.213	0.260	0.330	0.212
FSDBT	67.318	112.593	47.466	0.256	0.322	0.210
TSDBT	67.318	111.014	47.596	0.255	0.325	0.215
BSDBT	67.320	110.500	47.570	0.256	0.319	0.210
FE	67.557	110.808	47.527	0.256	0.327	0.216
Ref. [24]	67.103	110.322	47.520	0.255	0.326	0.215

**Table 6:** Comparison of dimensionless critical buckling loads obtained from different beam theories and FE simulations (*L*/*h* = 10).

BCs	*e*_0_	FGX					UD					FGO				
		CBT	FSDBT	TSDBT	BSDBT	FE	CBT	FSDBT	TSDBT	BSDBT	FE	CBT	FSDBT	TSDBT	BSDBT	FE
H-H	0.00	0.8225	0.8019	0.8019	0.8019	0.8020	0.8225	0.8019	0.8019	0.8019	0.8020	0.8225	0.8019	0.8019	0.8019	0.8020
	0.90	0.5547	0.5331	0.5210	0.5129	0.5119	0.3128	0.3049	0.3049	0.3049	0.3050	0.1684	0.1663	0.1667	0.1667	0.1655
	0.99	0.5279	0.5054	0.4856	0.4430	0.4097	0.2410	0.2349	0.2349	0.2349	0.2350	0.1030	0.1021	0.1022	0.1022	0.1024
H-C	0.00	1.6826	1.5908	1.5909	1.5909	1.5954	1.6826	1.5908	1.5909	1.5909	1.5954	1.6826	1.5908	1.5909	1.5909	1.5954
	0.90	1.1348	1.0400	0.9910	0.9600	0.9585	0.6398	0.6049	0.6049	0.6049	0.6067	0.3445	0.3352	0.3370	0.3366	0.3354
	0.99	1.0800	0.9816	0.9033	0.7584	0.6572	0.4929	0.4660	0.4661	0.4661	0.4674	0.2107	0.2066	0.2073	0.2072	0.2084
C-C	0.00	3.2900	2.9836	2.9840	2.9842	3.0004	3.2900	2.9836	2.9840	2.9842	3.0004	3.2900	2.9836	2.9840	2.9842	3.0004
	0.90	2.2188	1.9092	1.7637	1.6772	1.6738	1.2510	1.1345	1.1347	1.1347	1.1409	0.6736	0.6419	0.6478	0.6467	0.6464
	0.99	2.1116	1.7922	1.5666	1.2093	0.9847	0.9638	0.8741	0.8742	0.8742	0.8790	0.4119	0.3981	0.4005	0.4002	0.4040

**Table 7:** Comparison of dimensionless fundamental frequencies obtained from different beam theories and FE simulations (*L*/*h* = 10).

BCs	*e*_0_	FGX					UD					FGO				
		CBT	FSDBT	TSDBT	BSDBT	FE	CBT	FSDBT	TSDBT	BSDBT	FE	CBT	FSDBT	TSDBT	BSDBT	FE
H-H	0.00	0.2838	0.2802	0.2802	0.2801	0.2803	0.2837	0.2802	0.2802	0.2801	0.2803	0.2837	0.2802	0.2802	0.2801	0.2803
	0.90	0.2964	0.2907	0.2874	0.2850	0.2849	0.2228	0.2201	0.2201	0.2199	0.2201	0.1637	0.1627	0.1629	0.1628	0.1626
	0.99	0.3084	0.3019	0.2961	0.2829	0.2724	0.2088	0.2062	0.2062	0.2061	0.2062	0.1367	0.1361	0.1362	0.1362	0.1364
H-C	0.00	0.4429	0.4283	0.4285	0.4279	0.4294	0.4429	0.4283	0.4285	0.4279	0.4294	0.4429	0.4283	0.4285	0.4279	0.4294
	0.90	0.4626	0.4391	0.4273	0.4194	0.4188	0.3479	0.3363	0.3365	0.3361	0.3372	0.2556	0.2514	0.2522	0.2518	0.2523
	0.99	0.4814	0.4548	0.4338	0.3944	0.3608	0.3259	0.3151	0.3152	0.3149	0.3159	0.2135	0.2110	0.2115	0.2111	0.2123
C-C	0.00	0.6426	0.6054	0.6061	0.6061	0.6083	0.6426	0.6054	0.6061	0.6061	0.6083	0.6426	0.6054	0.6061	0.6061	0.6083
	0.90	0.6710	0.6127	0.5864	0.5710	0.5674	0.5046	0.4754	0.4760	0.4759	0.4777	0.3708	0.3600	0.3621	0.3618	0.3629
	0.99	0.6982	0.6325	0.5869	0.5131	0.4487	0.4728	0.4454	0.4459	0.4459	0.4475	0.3098	0.3033	0.3044	0.3044	0.3064

## Data Availability

The original contributions presented in the study are included in the article, further inquiries can be directed to the corresponding author.
